# Annotations

**Published:** 1907-08-03

**Authors:** 


### ANNOTATIONS.

### The President's Address.

The annual meeting of the British Medical Association at Exeter was welcomed by its President, Dr. Henry Davy, in a singularly graceful and pleasing address. After some allusions to the history of the city and to some of the more prominent local practitioners of bygone days, Dr. Davy remarked on the contrast between the present position of the Association, and that which it occupied when it last met in Exeter some sixty-five years ago. Extending a similar comparison to the development of medical knowledge he attributed the modern accuracy of observation and practice to the perfection of the microscope and to the development of organic chemistry. From these have arisen the discovery both of the actual causes of many diseases and an appreciation of the way in which these produce disturbances of the structure and functions of various parts of the body. At the same time it is necessary to recognise the practical value to the physician of much knowledge which is entirely empirical, and which, though not yet capable of scientific explanation, is highly useful in the treatment of disease. Even when the complete restoration of vigorous health is impossible much may be done by such treatment to relieve suffering and to prolong life. Dr. Davy strongly advocated the extension into public opinion of an appreciation of the value of physical culture as a means not only of promoting the health of the individual, but also of assisting the attainment of national efficiency. He considered that in the educational process necessary to secure this the medical profession should take an active and prominent part. In similar fashion he advised constant pressure on all educational authorities for the purpose of obtaining due recognition of the necessity of supervising the health and physical development of the children under their charge. And as a tribute to the value of preventive medicine and as an evidence of the services rendered by it to the public, he quoted the case of a practice to which fifty years ago typhoid fever contributed not less than £300 a year, while during the past few years the income from the same source hardly averaged five guineas per annum.

### The New Sydenham Society.

The announcement that the Council of this Society has determined to recommend, at the forthcoming annual meeting, that the Society be dissolved, will cause much regret, though this, we think, will not be largely associated with a sense of surprise. There is no question that during its existence the Society has done good work, and has rendered accessible to English readers numerous foreign medical works which otherwise would not have been available. But promptitude and business despatch have scarcely been the mark of the Society's proceedings, and thus it is not to be wondered at that it has failed to attract a larger measure of support. The want of effective management and control can be easily read in the statement which has been issued on behalf of the Council. From this it is evident that an unduly sanguine and optimistic spirit has been allowed to influence proceedings which ought to have been conducted on strictly business principles. The issue of the Clinical Atlas, it is acknowledged, was planned on too large a scale, and an expenditure was incurred far in excess of that which events have shown to be justifiable. Apparently, too, this new departure was undertaken when the responsibilities of the Society in connection with some of its earlier transactions were not fully discharged. Thus we are told that "for several years past the annual income has been to some extent burdened by debts incurred in former years," and an index of the methods of financial management permitted by the Council may be found in the statement that "it was impossible to allot to each year its own share in expenditure, and the auditors were repeatedly obliged to note that while the income and outgoings were balanced, there remained some outstanding accounts with artists, etc." A similar state of matters is not without parallel in certain other medical undertakings, and in it may be found a warning against an arrangement which, under the cover of a sonorous title, permits vague and unwarranted aspirations to lead an organisation away from the paths of steady and effective management and control.

### Opportunities for Medical Women.

In his recent address at the London School of Medicine for Women, Professor William Osler discussed the opportunities offered by the medical profession to women, and made no secret of the special difficulties and strain involved in general practice. On the other hand, he pointed out that there were several alternative branches of professional work in which the particular qualities possessed by women might find abundant scope. Among these was mentioned the public health and sanitary services, where, indeed, excellent work has already been done by women practitioners. A recent development at the Middlesex Hospital is likely to lead to increased activity in this direction. By means of a fund which has been specially raised for the purpose, the governing board of the hospital has been enabled to open well-equipped laboratories which are to be entirely devoted to the training of women practitioners in public health work and in the methods of bacteriological research. The department will be under the control of Mr. A. G. R. Foulerton, F.R.C.S., who will have as his assistant Miss Hilda Whittingham, M.B. This new development will be watched with great interest as completing the opportunities offered by the metropolis for the medical education of women in a specialised branch of practice. It must be recognised that the tendency of modern events is to create greater numbers of positions which demand expert sanitary and hygienic knnowledge, and for many of these women practitioners may reasonably offer themselves. Thus, in addition to an increasing demand for efficient medical officers of health, there are such positions as inspectors of factories and schools, and also administrative positions in connection with the Midwives Act. For the duties involved in these appointments a special technical training is advisable, if not essential.

